# No Evidence for Hepatitis E Virus Genotype 3 Susceptibility in Rats

**DOI:** 10.3201/eid1908.130200

**Published:** 2013-08

**Authors:** Tian-Cheng Li, Yasushi Ami, Yuriko Suzaki, Naokazu Takeda, Wakita Takaji

**Affiliations:** National Institute of Infectious Diseases, Tokyo, Japan (T.-C. Li, Y. Ami, Y. Suzaki, W. Takaji);; Osaka University Research Institute for Microbial Diseases, Osaka, Japan (N. Takeda)

**Keywords:** hepatitis E virus, rat HEV, rats, genotype 3, viruses, nude rats, rat study, susceptibility, immunodeficient, zoonoses

**To the editor:** Hepatitis E virus (HEV) is a positive-sense single-stranded RNA virus (genus *Hepevirus*, family *Hepeviridae*) (*1*). In humans, acute hepatitis infection caused by HEV is a serious public health concern in developing countries. Four HEV genotypes, G1–4, have been isolated from humans (*2*). G3 and G4 HEV have also been isolated from swine, wild boars, wild deer, and mongooses; these animals are thought to be the reservoirs of HEV (*3*). Direct evidence has indicated that HEV is transmitted from pigs or wild boars to humans; therefore, hepatitis E caused by G3 and G4 HEV infection is recognized as a zoonosis (*3*).

Although rats have long been suspected to be a potential reservoir for human HEV, no direct evidence has been found. The susceptibility of rats to human HEV genotypes is controversial. For example, anti-HEV IgG has been detected in various rat species, including Norway (*Rattus norvegicus*), black (*Rattus rattus*), and cotton (*Sigmodon hispidus*) rats, by using ELISA with antigens derived from G1 HEV. These results suggest that HEV or HEV-like virus infections occur in wild rats. However, the virus genome has not been detected, and the source of the infection was confirmed in few cases; thus far, it is not clear whether the anti-HEV IgG was induced by HEV or other HEV-like viruses. The detection of a partial genome of G1 HEV from wild rats in Nepal was reported in 2002 (*4*); however, this report was retracted in 2006 because the isolated strain was determined to be a result of laboratory contamination. Recently, Lack et al. isolated strains of G3 HEV from a variety species of wild rats in the United States (*5*); this finding suggests that wild rats are hosts for G3 HEV. Maneerat et al. also reported that human HEV (presumably G1) was transmissible to Wistar laboratory rats (*6*). However, Purcell et al. recently reported that G1, G2, and G3 do not infect laboratory rats (*7*), and we found in a previous study that laboratory rats are not susceptible to G1, G3, or G4 HEV (*8*). 

To further investigate the potential susceptibility of rats to infection with human HEV, we experimentally injected nude rats with G3 HEV and monitored virus growth. We used 2 samples of G3 HEV for the infection experiments, 1 derived from fecal specimens collected from a pig farm in Japan (GenBank accession no. DQ079632) and 1 derived from the supernatant of a hepatocarcinoma cell line, PLC/PRF/5, that was injected with the pig specimen. The infectivity of these samples was confirmed by experimental infections of cynomolgus monkeys (*9*; data not shown). 

Six 15-week-old female nude rats (athymic rats, Long-Evans-run/run; Japan SLC, Inc., Hamamatsu, Japan) were used in this study. These rats, which are bred to be immunodeficient, are known to be susceptible to rat HEV, but it is unknown if they are susceptible to other types of HEV. All rats were negative for G3 HEV RNA and anti-HEV antibodies, as determined by nested reverse transcription PCR (*10*) and ELISA (*8*), respectively. Rats were housed individually in biosafety level 2 facilities. Experiments were reviewed by the ethics committee of the National Institute of Infectious Diseases (NIID) Japan and carried out according to the “Guidelines for animal experiments performed at NIID” under code 113060. 

The 6 rats were randomly assigned to 2 groups, injected intravenously with 500 µL of an HEV sample suspension through the tail vein, and monitored for 3 months. The 3 rats in group 1 were injected with the sample derived from pig feces, which contained 5 × 10^4^ copies of G3 HEV; the 3 rats in group 2 were injected with the cell culture supernatant sample, which contained 4 × 10^6^ copies of G3 HEV. Serum samples were collected weekly for examination of HEV RNA and anti-HEV IgG and IgM and were also used to determine alanine aminotransferase values. Fecal samples were collected every 3 days to detect HEV RNA. The animals were humanly killed by exsanguination 91 days postinjection, liver tissues were collected, and a 10% tissue suspension was prepared as described (*8*).

For groups 1 and 2, all serum samples collected 1–13 weeks postinjection were negative for HEV RNA and anti-HEV IgG and IgM. HEV RNA also was not detected in fecal samples or liver tissues. Alanine aminotransferase elevation was not observed in any serum samples.

In conclusion, even by using samples with high titers of HEV RNA in injection experiments, we were unable to cause infection with G3 HEV in immunodeficient nude rats. We found no evidence that rats are susceptible to infection with G3 HEV.

**Table:** 
